# The role of omental transposition for the management of postoperative mediastinitis: a case series

**DOI:** 10.1186/1757-1626-2-142

**Published:** 2009-02-23

**Authors:** Panagiotis Hountis, Panagiotis Dedeilias, Konstadinos Bolos

**Affiliations:** 1 Evaggelismos Hospital, Department of Cardiac Surgery, Ipsilantou 45-47, Athens, Greece

## Abstract

**Introduction:**

The aim of our study is to present our experience from the management of six patients with deep sternal wood infection and mediastinitis after aortocoronary by pass grafting.

**Case series:**

Five Caucasian Greek male patients and a Caucasian Greek female were subjected to aortocoronary by pass grafting. Mean time of sternal dehiscence and mediastinitis was 9–17 (mean 11) days. We managed these patients with total sternectomy and transposition of the greater omentum in the thorax. All patients had an uneventful postoperative course.

**Conclusion:**

We believe that greater omentum is the ideal reconstruction tissue for deep sternal wound infections and mediastinitis. Timely diagnosis, aggressive sternal debridement and omental flap coverage represent the mainstay of therapy in this highly lethal complication.

## Introduction

Median sternotomy was first described by Milton in 1887 and is considered the most usually performed incision in cardiac operations. Although newer techniques aim at smaller and minimal invasive chest operations, median sternotomy has many advantages, it can be performed fast, easy and with little if any blood loss. The most important complication of median sternotomy is the infection of the surgical incision that may lead to sternal dehiscence and osteitis, osteomyelitis and mediastinitis development. Median sternotomy disruption and mediastinitis is a rare complication (0,3–5%) that has been associated with high mortality rates. (14–40%) The main etiologic factors that have been implicated in this complication is obesity, diabetes mellitus, chronic obstructive pulmonary disease, the length of the operation and high volume of blood loss.[1,2]

## Case series presentation

During the years 2003–2007, 768 patients were subjected to aortocoronary by pass grafting from our department. Six (6) patients developed sternal disruption and mediastinitis. (0,8%). The basic parameters of the patients' history and operative details can be seen on Table 1.

**Table 1:** Perioperative characteristics of the patients

Sex/Age	Predisposing conditions	Operation	Surgical parameters	Infection characters	Microbiology	ICU and Hospital stay
male 1 60	DM, obesity, smoking	3CABG, 1IMA	55 min pump time 210 min operative time 3 blood units	9^th ^day, fever, pain, drainage of fluid from sternum	Staphylococcus aureus	ICU 4 days Hospital stay 20 days
male 2 61	DM, obesity, smoking	3CABG, 1IMA	40 min pump time190 min operative time 2 blood units RBC	9^th ^day, fever, drainage of fluid from sternum	Staphylococcus aureus Staphylococcus epidermidis	ICU 9 days Hospital stay 19 days
male 3 65	DM, obesity, smoking, chest reopening for hemorrhage	3CABG, 1IMA	60 min pump time 170 min operative time 10 blood units RBC	10^th ^day in ICU, fever	Staphylococcus aureus Staphylococcus epidermidis	ICU 12 days Hospital stay 27 days
male 4 69	DM, obesity, smoking, chest reopening for hemorrhage, Chronic renal failure	3CABG, 1IMA	50 min pump time 220 min operative time 5 blood units RBC	11^th ^day, pain, red incision, mental status problems	Staphylococcus aureus	ICU 12 days Hospital stay 31 days
male 5 74	DM, obesity, smoking, chest reopening for hemorrhage, COPD	4CABG, 1IMA	65 min pump time150 min operative time 8 blood units RBC	12^th ^day in ICU, fever, pain, pus from the incision	Pseudomonas aeroginosa	ICU 21 days Hospital stay 29 days
female 62	DM, obesity, extremely big breasts, chest reopening for hemorrhage	2CABG, 1IMA	35 min pump time 130 min operative time 2 blood units RBC	17^th ^day, weakness, fluid drainage from the sternum, pain	Pseudomonas aeroginosa	ICU 10 days Hospital stay 46 days

DM: Diabetes Melitus

CABG: Coronary artery by pass grafting

IMA: Internal mammary artery

RBC: Red Blood Cells

ICU: Intensive Care Unit

These patients were five (5) men 60–74 year old and one (1) female 62 year old, all Greek Caucasians. All the patients were diabetics and obese. Their postoperative course was initially normal. The mean time of sternal disruption and the development of mediastinitis was eleven (11) (9–17) days. The main presenting symptoms were high fever and generalized septic condition. Microbiology exam of the sternal and substernal tissues showed that staphylococcus aureus and staphylococcus epidermidis was the main pathogens in four (4) of the patients and pseudomonas aeroginosa in two (2).

**Figure 1:**
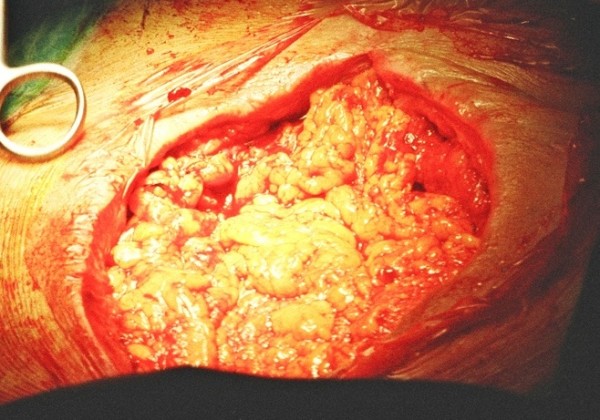
**Dissection of the greater omentum alomg with the right gastroepiploic artery**.

**Figure 2:**
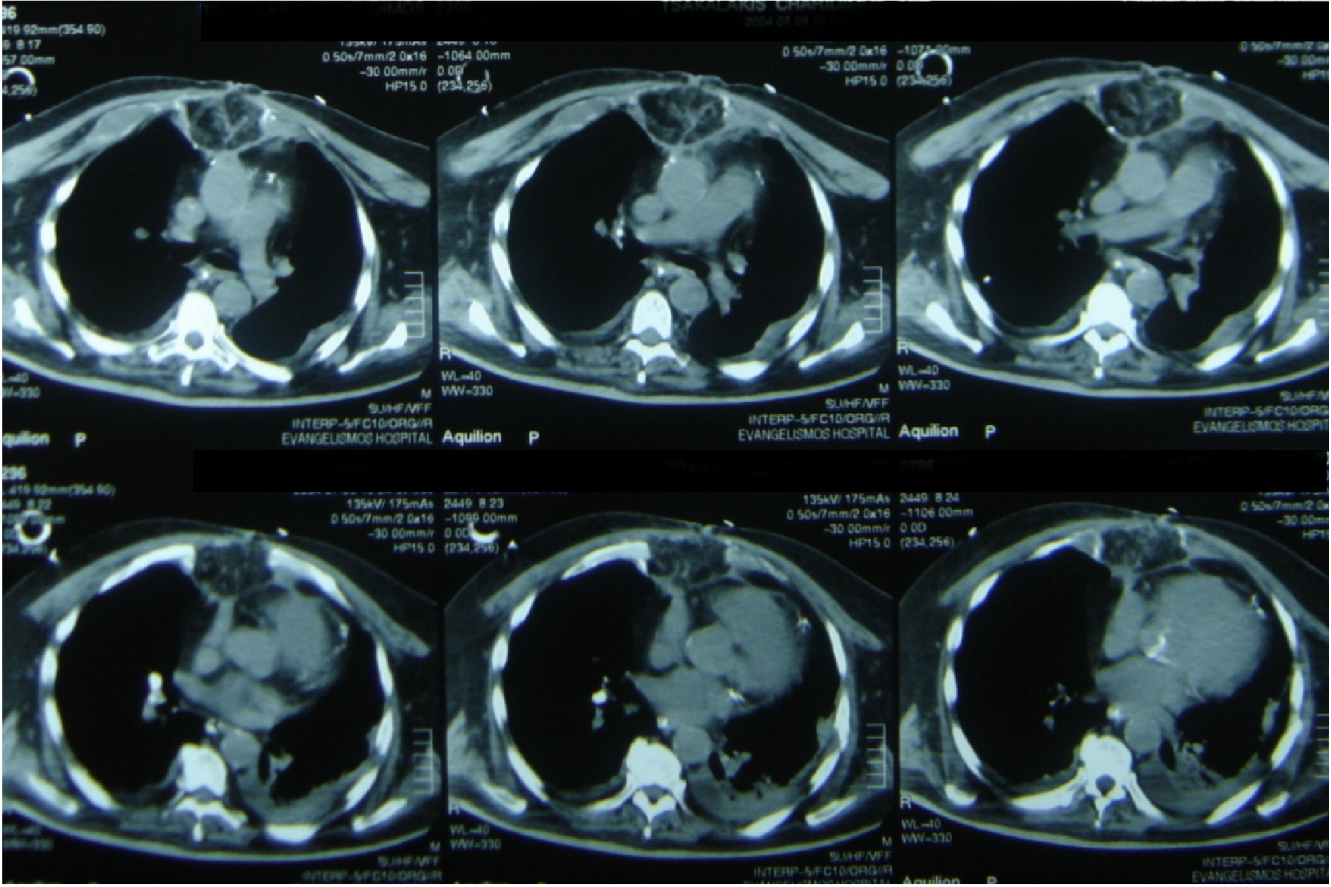
**Result of the omental transfer one year after the operation in a chest CT scan**.

The management of these cases was conducted in two stages. First, we opened the chest and we removed all the wires and any other foreign material from the pericardial cavity. We took multiple cultures from different areas of the pericardial cavity. At the same time we cleaned the area with irrigation and all the necrotic and inflammatory tissues and debris. The cleaning of the cavity with irrigation was done three times a day with antiseptic solutions and it was kept open with sterilized pads inside. We implemented a system of continuous 24 hour automated irrigation of a solution with antibiotic (Vancomycin), normal saline and antiseptic solution (povidone iodine). This was proved to be effectively by removing necrotic tissues by irrigation and providing effective antiseptic coverage. Blood and urine cultures were taken every day from the patients. We performed these actions from 10 to 21 days depending on the macroscopic picture of the pericardial cavity and the results of three consecutive pericardial cultures that should be negative. At the second stage we performed an operation in order to close the open chest. Intraoperatively we removed the sternum completely and some portions of the chondral part of the ribs of the thorax. With a small laparotomy we dissected the greater omentum alomg with the right gastroepiploic artery and we transferred this into the pericardial cavity in order to eliminate the dead space. (Figure 1) 1. In Figure 2 2 we can see the result of the omental transfer one year after the operation in a chest CT scan.

All the patients survived with these techniques and the mean hospital stay was 28.6 days (20–46). Six months, one year and two years follow up showed that the patients were in good clinical condition free of symptoms.

## Discussion

Mediastinitis is a devastating potential complication of cardiac surgery. Although the rate of incidence in patients who have undergone a median sternotomy for cardiac surgery with cardiopulmonary bypass (CPB) is low (from 1% to 2.5%) the associated mortality rate varies from 14% to 47%.

The exact mechanism by which mediastinitis develops is unknown and multifactorial. Intraoperative wound contamination has been conclusively demonstrated in a small number of cases and probably represents an important source of many infections. In addition, a variety of patient characteristics have been associated with an increased incidence of mediastinitis, suggesting that certain factors may predispose patients to the development of this complication.[3]

The role of the omentum in containment of abdominal infections is well recognized. A relatively long vascular pedicle enables omental transfer to the anterior mediastinum as is needed for post-sternotomy mediastinitis. [4]The omentum is known to be rich in lymphatic and blood vessels that it can absorb inflammatory exudate rapidly and prevent further extension of local infection.

The importance of prompt diagnosis of postoperative mediastinitis and emergent operation must be stressed in these cases. Any delay in making the diagnosis and surgical treatment often results in septic shock followed by multiple organ failure or fatal hemorrhage from the surgical suture line on the heart or great vessels. Even if the symptoms are not indicative of mediastinitis, even with the suspect of this complication emergent chest CT has been recommended. Spiky high fever after an afebrile postoperative period, even without any wound signs, or leukocytosis are the main presenting alarm signs in these cases.[5,6]

## Conclusion

Mediastinitis after cardiac or thoracic surgery is a serious complication with major implications regarding morbidity and mortality. Aggressive early debridement, open wound and continuous high volume irrigation of the wound has proved to be very beneficial for the patients. Although the numders are small we believe that our technique is highly effective. The 100% survival after this highly fatal complication suggests that this technique is the most suitable option for the treatment of postoperative mediastinitis. We would like to emphasize that a high clinical index of suspicion is extremely important in early diagnosis and management of sternal dehiscence and postoperative mediastinitis.

## Consent

"Written informed consent was obtained from the patient for publication of this case report and accompanying images. A copy of the written consent is available for review by the Editor-in-Chief of this journal."

## Competing interests

The authors declare that they have no competing interests.

## Authors' contributions

PH analyzed and interpreted the patient data. PD was a major contributor in writing the manuscriptK. B was a major contributor in writing the manuscript.

All authors read and approved the final manuscript.
